# Utilization of emergency ophthalmology services in Taiwan: a nationwide population study

**DOI:** 10.1038/s41598-020-74815-1

**Published:** 2020-10-19

**Authors:** Min-Huei Hsu, Chia-An Hsu, Sheng-Huang Hsiao, Dachen Chu, Ju-Chuan Yen

**Affiliations:** 1 Graduate Institute of Data Science, College of Management, Taipei Medical University, Taipei, Taiwan; 2 Department of Neurosurgery, Wan-Fang Hospital, Taipei Medical University, Taipei, Taiwan; 3 Department of Medical Education, Taipei Veterans General Hospital, Taipei, Taiwan; 4 Department of Neurosurgery, Ren-Ai Branch, Taipei City Hospital, Taipei, Taiwan; 5 National Chengchi University, Taipei, Taiwan; 6 Department of Education and Research, Taipei City Hospital, Taipei, Taiwan; 7 Department of Ophthalmology, Ren-Ai Branch, Taipei City Hospital, Taipei, Taiwan; 8 Graduate Institute of Biomedical Informatics, College of Medical Science and Technology, Taipei Medical University, Taipei, Taiwan

**Keywords:** Eye diseases, Diagnosis, Public health

## Abstract

The aim of this study was to conduct a nationwide survey of the use of emergency ophthalmology services using a sub-dataset of one million beneficiaries sampled from Taiwan’s National Health Insurance Research Database (NHIRD) for the years 2008 through 2012. By analyzing this population dataset, the study illustrates the disease landscape of emergency eye care services. The five-year, one-million-person NHIRD sub-dataset for 2008 through 2012 was used to explore emergency visits and ophthalmology specialty visits and to analyze the associated demographics and diagnosis codes based on the International Classification of Diseases, 9th Revision, Clinical Modification (ICD-9-CM). Diagnoses were categorized into three groups: urgent, non-urgent, and intermediate. A total of 2454 emergency eye care visits were identified. The mean age of the patients who made these visits was 34.6 years old, and their sex ratio was 1.36 men to women. The percentages of urgent, non-urgent, and intermediate eye care visits in this study were 48.2%, 30.9%, and 20.9%, respectively. The leading diagnoses in the urgent category were corneal abrasions, foreign bodies in the eyes, eye burns, and blunt eye injuries. The leading diagnoses for the non-urgent visits were conjunctivitis, subconjunctival hemorrhages, trichiasis, and dry eye disease. Those for the intermediate category were superficial punctate keratitis, corneal opacity and degeneration, and lid, orbital, and lacrimal drainage infections. The urgent visit category accounted for nearly half of all the visits identified in this study. Compared to outpatient department visitors, the emergency ophthalmology service patients were younger and more predominantly male. These results were consistent with those of previous reports. Low copays have made emergency ophthalmology services highly accessible in Taiwan. However, future policies can be designed to more effectively allocate resources to urgent cases.

## Introduction

Eye care services provided in emergency departments are of critical importance to the visual health of entire populations^1^. Devastating conditions, such as eyeball ruptures or retinal detachments, are often triaged and treated in emergency departments. The accurate diagnosis and timely management of such vision-threatening conditions play pivotal roles in the visual outcomes of the patients affected by them.

Taiwan launched its National Health Insurance (NHI) program on March 1st, 1994. Over 99% of the residents of Taiwan are covered by the program. The health care delivery system in Taiwan is mostly supported by this single-payer mandatory insurance system. Three departments, including the inpatient department, the outpatient one and the emergent service made up the health care delivery system in Taiwan. To most of the beneficiaries, the out of pocket registration fee for outpatient visits ranged from 50 to 420 New Taiwan Dollars (NTD) according to level of the institution. As for the medication fee, 200 NTD is the upper limit for each visit and the examination fee is entirely covered by the NHI program. In terms of emergent services, the out of pocket fee ranged from 150 to 550 NTD. Lastly, the out of pocket fee of inpatient service ranged from 5 to 30% of the total cost according to the duration of stay and the type of ward. The miniscule out of pocket pay made all three departments accessible to most of the population.

In terms of the eye care services provided by the system, until 2020, there are 1958 registered ophthalmologist according to the registry provided by the government, which is about 85 ophthalmologists per million population. This is higher than the estimate of 76.2 ophthalmologist in high-income countries according to previous study^2^. Moreover, the beneficiaries of the NHI are free to visit all specialists at will, including the ophthalmologists. No referral from primary care providers are required for a visit to the eye care service. The average frequency of visit is 0.7 visits per year according to our previous work.

In Taiwan, the eye care services provided by emergency departments start with an evaluation conducted by an emergency physician^3,4^. An ophthalmologist only becomes involved when such involvement is deemed necessary by the attending emergency physician^5^. Due to the limited number of consulting ophthalmologists available, it is critical that the timing of their involvement is optimized to the extent possible.

That is, in order to efficiently triage and manage patients, knowledge of the disease landscape for emergency eye care services is vital^6^. A previous study describing the utilization of emergency department ophthalmology services in the United States based on nationwide data showed that only 41.2% of the visits were truly urgent^7,8^. Furthermore, while another study described an attempt to use visual acuity as an indicator of ocular emergencies, that study did not utilize a nationwide survey^9,10^. In Taiwan, however, the National Health Insurance Research Database (NHIRD) allows for a nationwide survey of the disease landscape of emergency eye care services.

The aim of this study was to conduct a nationwide survey of the use of emergency ophthalmology services using a sub-dataset of one million beneficiaries sampled from Taiwan’s NHIRD for the years 2008 through 2012. By analyzing this population dataset, the disease landscape of emergency eye care services in Taiwan can be illustrated. The reported epidemiological factors of emergency eye care services can then serve as a reference of critical value to emergency physicians and ophthalmologists.

## Materials and methods

This study was approved by the institutional review board of Taipei Medical University.

### Data source: the one-million-person sub data set of the NHIRD

For research purposes, the NHI administration has released claims data for the program since 1996. Among this data is the one-million-person sub-dataset of the NHIRD for the years 2008 through 2012. This one-million-person sub-dataset includes all the claims data from 2008 through 2012 for one million beneficiaries who were randomly selected from the larger NHIRD. There are no significant differences in age, sex, or average-insured payroll-related premiums between the sample group and the larger population of all NHI enrollees.

### Data processing

Visits for emergency eye care services were extracted from the one-million-person sub-dataset. The code for emergency department services (02) was first used to extract all emergency department visits. The eye care services were then extracted using the specialty code for ophthalmology (10). The associated demographics, costs, and International Classification of Diseases, 9th Revision, Clinical Modification (ICD-9-CM) codes of the diagnoses were then retrieved and parsed using descriptive analytics.

The ICD-9-CM codes were categorized into emergent, non-emergent, and intermediate groups. This framework of categorizing patients according to the degree of emergency had been done before. However, we modified the framework to better suit the eye care service in Taiwan. The urgent group codes were defined as those for vision-threatening, acutely painful conditions such as open lid or open globe injuries. The non-urgent group codes were defined as those for non-vision-threatening and not acutely painful situations such as subconjunctival hemorrhages. The intermediate group codes included those for diseases and conditions with borderline outcomes, such as superficial punctate keratitis.

### Statistical analysis

SAS for Windows 9.4 (SAS Institute, Inc., Cary, NC, USA) was used to perform descriptive statistical analyses. Chi-square test and independent t-test was performed with significant alpha level of 0.05.

## Results

### Demographics of emergency eye care services

A total of 1,328,725 emergency visits with the emergency department code were extracted from the dataset. Of these visits, 2454 (0.18%) of them were labelled with the specialty code of 10 for ophthalmology. The mean number of such visits per year was 490.8 visits. The mean age of the patients who made these visits was 34.6 years old, with a standard deviation of 19.7 years, and the age range was from 0 to 97. Of all those patients, 42.4 percent were female. The sex ratio was thus 1.36 men to women.

### The epidemiology of emergency eye care services

During the studied period, urgent diagnoses codes were assigned for 48.2% of the visits, while non-urgent codes were assigned for 30.9% of the visits and intermediate group codes were assigned for the remaining 20.9% (Table 1). The four most frequent diagnoses among the urgent diagnoses were foreign bodies in the eyes (15.0% of all visits), corneal abrasions (13.3%), burns to the eyes and adnexa (4.0%), and blunt eye injuries (3.8%). Conjunctivitis (24.8%), subconjunctival hemorrhage, (5.0%), dry eye disease (0.4%), and trichiasis (0.4%) were the most common reasons for the non-urgent visits, while superficial punctate keratitis (9.5%) and corneal opacity and degeneration (6.1%) were the most frequent causes for the intermediate group visits.

**Table 1:** The incidence of diagnoses by urgency category.

Diagnoses	Percentage, n (%)
**Urgent diagnoses**	
Foreign body of conjunctiva or cornea	376 (15.3)
Corneal abrasion	326 (13.3)
Burn of eyes and adnexa	98 (4.0)
Blunt eye injury	93 (3.8)
Corneal ulcer	71 (2.9)
Glaucoma	61 (2.5)
Open wound of lid and eye	59 (2.4)
Retinal disorders	52 (2.1)
Uveal disorders	25 (1.0)
Globe disorder	10 (0.4)
Herpes	6 (0.25)
Orbital floor fracture	3 (0.14)
Optic nerve lesion	2 (0.1)
Total	1182 (48.2)
**Intermediate diagnoses**	
Superficial punctate keratitis	234 (9.5)
Corneal opacity & degeneration	120 (4.9)
Lid orbit lacrimal drainage infection	83 (3.4)
Other systemic symptoms	49 (2.0)
Others	27 (1.1)
Total	513 (20.9)
**Non-urgent diagnoses**	
Conjunctivitis	609 (24.8)
Subconjunctival hemorrhage	123 (5.0)
Trichiasis	10 (0.4)
Dry eye disease	10 (0.4)
Chalazion	7 (0.3)
Total	759 (30.9)

### Demographics of the urgent diagnoses group

**Figure 1:**
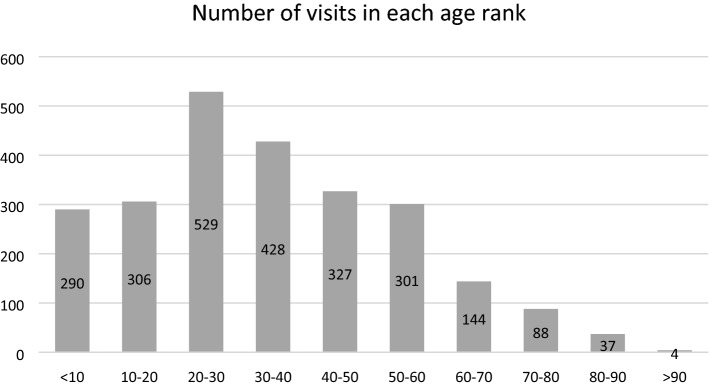
Number of visits in each age rank.

The age distribution among all patients revealed a central peak among people aged 20 to 40 years old (Fig. 1). Independent t-test and chi-square test was applied to evaluate the significance of the difference among age groups and between sex. There was significant difference of age between patients with urgent diagnoses code and that of the patients with non-urgent or intermediate diagnoses code (p < 0.001). However, no significant correlation between sex and severity was found (p = 0.0780) (Table 2).

**Table 2:** Distribution of diagnosis code severity among age and sex groups.

	Non-urgent and intermediate	Urgent	p-value
	Mean (SD)	Mean (SD)	
Age	32.4 (20.2)	37.3 (18.7)	< 0.001^1^

	N	N	
**Sex**			
F	558	476	0.0780^2^
M	714	706	

^1^Independent t-test.

^2^Chi-square test.

### Costs of the emergency eye care services

The median cost of all the emergency department eye care service visits was 692 New Taiwan Dollars (NTD) (interquartile range: 837 NTD), and the mean copay was 321 NTD with a standard deviation of 138 NTD. The diagnosis codes with costs within the first quartile (> 2000NTD) were further extracted and are listed in Table 3.

**Table 3:** Top quartile cost diagnoses.

> 2000 NTD diagnosis	Code ICD-9-CM
Open wound of eyeball and lids	871, 870
Retinal disorder	361, 362
Globe disorder	360
Glaucoma	365
Blunt eye injury	921
Optic nerve disorders	377
Uveal disorder	364
Corneal ulcer	37,000
Orbital floor fracture	802
Burn of eye and adnexa	940
Herpes zoster ophthalmicus	053

## Discussion

### Utilization of Taiwan’s emergency eye care services

According to previous research, ophthalmologic emergencies account for 3% of visits to emergency departments in the United States. However, such emergencies only accounted for around 0.2% of the visits for the population investigated in the current study. This is likely associated with the easily accessed ophthalmology outpatient service. Since no referral from primary care providers was required and the out of pocket registration fee was lower (50 to 420 NTD) compared to the emergency service (150 to 550 NTD). The beneficiaries were more motivated to visit the ophthalmologists’ outpatient clinic when the clinic is available for most scenarios.

### Comparison with outpatient department visits

For the outpatient visits extracted from the same sub-dataset, the mean age of the patients was 36.2 years old, and the male-to-female gender ratio was 0.764. In contrast, the emergency eye care visits had a mean patient age of 34 years old and a male-to-female gender ratio of 1.36. This reversed gender ratio might have been due to work-related injuries such as those from corneal foreign bodies and blunt eye injuries such as those from physical fights. Both types of injuries are more common among males^10^.

### Comparison of epidemiology, demographics, and costs of emergency eye care services with previous reports

This study reported results similar to those of previous reports from previous studies in terms of the degree of emergency eye care services for urgent reasons as determined by categorizing ICD-9-CM disease codes. Around half of the visits identified in this study were for urgent reasons (48.2%), while a previous study reported that 41.2% of eye care service visits in the United States^6^ were for urgent reasons. The demographics of the patients were also similar. The mean age of such patients in the United State was previously reported to be 31 years, while this study reported a mean patient age of 34. The male predominance of the patients in this study was also consistent with that previously reported for such patients in the United States^6^.

The leading diagnoses for the urgent visits in this study were corneal abrasions, foreign bodies in the eyes, eye burns, and blunt eye injuries. These were similar to the leading causes for eye care services previously reported for the United States, except that blunt eye injuries were reported to be more common than eye burns in the United States. The most common causes of non-urgent eye service visits were conjunctivitis and subconjunctival hemorrhages in both Taiwan and the United States^6^.

Other studies also reported high rate of non-emergent visits to the emergent eye care services. One study from Brazil reported of emergency eye care at a tertiary referral center that 69 percent of the cases only required simple treatment^11^. Another report from Iran reported similar mean age of 33.2 years old. Moreover, the study also reported male predominance in the studied patient population, which was likely related to the high percentage of eye trauma patients^12^. Still another study from a university referral center at United States also reported that over one-third of the patients are non-urgent and the non-urgent cases are related to female gender and the form of insurance coverage^13^.

Comparing to previous studies, our report of non-urgent dominance of diagnosis code, male predominance in the group of urgent diagnosis were consistent with previous studies. The low out of pocket payment might also contributed to the high non-urgent visits.

### Low cost and high accessibility of Taiwan’s emergency eye care services

The costs of emergent eye care services were considerably lower in Taiwan than in the United Stated, with a median cost of 692 NTD (around 23 USD) for an emergency visit and a mean copay of 321 NTD (around 11 USD). The median costs of emergency eye care visits in the United States were more than 1000 USD for urgent cases and 600 to 800 USD for non-urgent cases. The relatively low copays for such services in Taiwan might be the cause of the high proportion of emergency eye care service visits for non-urgent reasons.

Diagnoses resulting in high costs, such as open wounds to the lids and eyeballs, glaucoma, globe disorders, and retinal lesions (diagnoses codes with costs in the top quarter listed in Table 3), reflected authentic ophthalmic emergency eye care visits. Future policies can be designed to more effectively allocate resources for the management of patients with such conditions. At the same time, NHI beneficiaries should be discouraged from visiting emergency departments with non-urgent complaints. For example, copays could be made higher for visits made for non-urgent reasons in order to reduce the overall burden placed on emergency eye care services.

## Conclusions

A one-million-person sub-dataset of Taiwan’s NHIRD covering 5 consecutive years was used to investigate emergency ophthalmology visits in Taiwan. The demographics showed that the mean age of patients in such visits was 34.6 years, and that the patients were predominantly male. The costs and copays for these emergency ophthalmology visits were low compared to those for such visits in the United States. The leading diagnoses for urgent, non-urgent, and intermediate categories were illustrated. The diagnoses codes with costs in the top quartile paralleled authentic emergency diagnoses. Future policies could be designed to encourage more effective allocation of resources for such truly urgent patients.

## References

[1] Lau JT, Lee V, Fan D, Lau M, Michon J (2004). Attitudes towards and perceptions of visual loss and its causes among Hong Kong Chinese adults. Clin. Exp. Ophthalmol..

[2] Resnikoff S (2020). Estimated number of ophthalmologists worldwide (International Council of Ophthalmology update): Will we meet the needs?. Br. J. Ophthalmol..

[3] Hsu CA, Hsiao SH, Hsu MH, Yen JC (2020). Utilization of outpatient eye care services in Taiwan: A nationwide population study. J. Ophthalmol..

[4] Yen JC, Lin HL, Hsu CA, Li YC, Hsu MH (2015). Atrial fibrillation and coronary artery disease as risk factors of retinal artery occlusion: A nationwide population-based study. Biomed. Res. Int..

[5] Bhatt R, Sandramouli S (2007). Evidence-based practice in acute ophthalmology. Eye (Lond).

[6] Channa R (2016). Epidemiology of eye-related emergency department visits. JAMA Ophthalmol..

[7] Wang SY, Hamid MS, Musch DC, Woodward MA (2018). Utilization of ophthalmologist consultation for emergency care at a University Hospital. JAMA Ophthalmol..

[8] Sagoo MS, Raina J (2009). Evidence-based medicine audit as a tool for improving emergency ophthalmology. Eye (Lond.).

[9] Babineau MR, Sanchez LD (2008). Ophthalmologic procedures in the emergency department. Emerg. Med. Clin. N. Am..

[10] Chang CH (2008). Hospitalized eye injury in a large industrial city of South-Eastern Asia. Graefes Arch. Clin. Exp. Ophthalmol..

[11] Carvalho Rde, S. & Jose, N. K. Ophthalmology emergency room at the University of Sao Paulo General Hospital: A tertiary hospital providing primary and secondary level care. *Clinics (Sao Paulo) ***62**, 301–308, 10.1590/s1807-59322007000300015 (2007). 10.1590/s1807-59322007000300015 17589671

[12] Jafari AK (2012). Different causes of referral to ophthalmology emergency room. J. Emerg. Trauma Shock.

[13] Samoilă O, Ostriceanu S, Samoilă L (2016). Epidemiology of ocular emergencies in Cluj ophthalmology clinic. Rom. J. Ophthalmol..

